# Time-resolved AUSAXS at BL28XU at SPring-8

**DOI:** 10.1107/S1600576723011135

**Published:** 2024-02-01

**Authors:** Yohei Nakanishi, So Fujinami, Motoki Shibata, Tsukasa Miyazaki, Katsuhiro Yamamoto, Mikihito Takenaka

**Affiliations:** a Institute for Chemical Research, Kyoto University, Gokasho, Uji, Kyoto 611-0011, Japan; b Office of Society-Academia Collaboration for Innovation, Kyoto University, Yoshida-Honmachi, Kyoto 606-8501, Japan; c Department of Life Science and Applied Chemistry, Graduate School of Engineering, Nagoya Institute of Technology, Nagoya 466-8555, Japan; Argonne National Laboratory, USA

**Keywords:** anomalous ultra-small-angle X-ray scattering, time-resolved measurements, channel-cut compact monochromators

## Abstract

An anomalous ultra-small-angle X-ray scattering (AUSAXS) system has been constructed at BL28XU at SPring-8 for time-resolved AUSAXS experiments. The path length was extended to 9.1 m and a minimum of *q* = 0.0069 nm^−1^ was attained. Scattering profiles at 0.0069 to 0.3 nm^−1^ were successfully obtained at 17 different X-ray energies in 30 s using the BL28XU optical setup, which enables adjustment of the energy of the incident X-rays quickly without the beam position drifting. Time-resolved measurements were conducted to investigate changes in the structure of zinc compounds in poly(styrene-*ran*-butadiene) rubber during vulcanization. A change in energy dependence of the scattered intensity with time was found during vulcanization, suggesting the transformation of zinc in the reaction.

## Abstract 2

This work reports the successful construction of an anomalous ultra-small-angle X-ray scattering (AUSAXS) measurement system at the BL28XU beamline at SPring-8. A minimum of *q* = 0.0069 nm^−1^ was attained and time-resolved measurements were carried out by performing scattering measurements at 17 different energies in 30 s.

## 1. Introduction

Anomalous small-angle X-ray scattering (ASAXS) is a contrast variation scattering technique that enables estimation of the partial structure factors in a multi-component system (Lyon & Simon, 1987 ▸; Naudon, 1995 ▸; Stuhrmann, 2007 ▸; Hoell *et al.*, 2009 ▸). In an ASAXS experiment, we measure the change in the scattered intensity with incident X-ray energy near the absorption edges of one component in the multi-component system. Near absorption edges, the scattering length or contrast factor of X-rays drastically changes (Cromer & Liberman, 1981 ▸). The analysis of the scattered intensity variation with the incident X-ray energy yields each partial structure factor of the multi-component system. For example, in the case of rubber-filler systems with sulfur and zinc oxide (ZnO), the *K*-absorption edge of Zn is used for ASAXS. Watanabe *et al.* (2023 ▸) applied ASAXS to poly(styrene-*ran*-butadiene) (SBR)/carbon black (CB) systems vulcanized with sulfur and ZnO, and obtained the partial structure factors of CB and Zn separately. The results revealed that the CB aggregates comprise closely packed CB primary particles. Watanabe and co-workers also found large particles of ZnO and particles of ZnS on the order of 10 nm. However, in the previous ASAXS experiments, the minimum wavenumber *q* = 0.01 nm^−1^ was not sufficient to analyze the network structure of the aggregation of CB, and it was difficult to conduct time-resolved ASAXS measurements quickly since it took several tens of seconds to adjust the X-ray energy of the incident beam. Thus, we have constructed an anomalous ultra-small-angle X-ray scattering (AUSAXS) system at BL28XU at SPring-8 for time-resolved AUSAXS experiments. At BL28XU, it is possible to quickly adjust the energy of the incident X-rays without the beam position drifting (Tanida *et al.*, 2014 ▸). We also extended the path length to 9.1 m and attained a minimum *q* = 0.0069 nm^−1^ at 9 keV. This laboratory note details the setup that enables us to conduct time-resolved AUSAXS measurements at BL28XU.

## 2. Basic concept

The optical system was designed to observe the scattered intensity at 0.005 < *q* < 0.3 nm^−1^ to evaluate submicrometre-scale structures. We also aimed to construct an experimental system in which AUSAXS measurements for the absorption edge of a specific element can be completed in less than 1 min to allow *in situ* follow-up of structural changes. In the case of typical vulcanization processes in rubber materials, it takes about 30 min to complete the vulcanization. Thus, AUSAXS measurements must be designed to be possible within 1 min.

## 3. Optical setup

**Figure 1:**
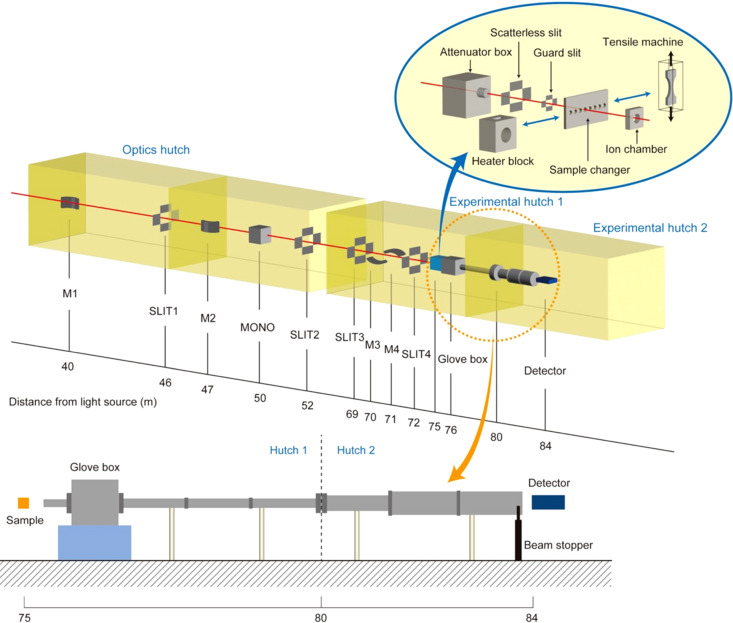
Schematic illustration of the BL28XU beamline.

Fig. 1 ▸ shows a schematic of the AUSAXS equipment for BL28XU. BL28XU consists of an optics hutch, experimental hutch 1 and experimental hutch 2. For AUSAXS measurements, we employed the existing optical setup for X-ray diffraction and X-ray absorption fine-structure measurements. The optical setup includes a first mirror (M1), a second mirror (M2), a monochromator (MONO) and two jaw slits (SLIT1 and SLIT2) in the optics hutch, and a third mirror (M3), a fourth mirror (M4) and two further jaw slits (SLIT3 and SLIT4) in experimental hutch 1. The details of each component are described by Tanida *et al.* (2014 ▸). The setup enables us to change the energy of the X-ray with the beam position at the focal point remaining constant. In addition to the existing optical setup, we installed new components for the AUSAXS setup, consisting of an attenuator box, scatterless slit and guard slit, as shown in Fig. 1 ▸. The attenuator box is located on the front side of the scatterless slit, with selectable attenuators of Al 0.1–0.5 mm, Mo 0.06–0.18 mm and Cu 0.1–0.4 mm, and we can adjust the attenuation ratio of the incident beam in the range 1–10^−40^ at an X-ray energy of 9 keV. The scatterless slit reduces parasitic scattering from upstream. The guard slit is immediately placed at the front of the sample position to reduce air-scattering. The X-ray beam size at the sample position is 100 µm (horizontal) × 100 µm (vertical) at 9 keV.

An automatic *XZ* stage is installed at the sample position, and we can measure up to eight samples using the sample changer with the *XZ* stage at room temperature. We are planning to install a vacuum chamber for the sample changer to reduce the background scattering. The sample changer can be replaced with a heater block, which allows the sample to be heated to 200°C, and a tensile machine for *in situ* observations during heating and stretching of samples. The ion chamber is mounted behind the sample to measure the transmittance of samples. A glove box placed behind the ion chamber is used for part of the flight path in the AUSAXS setup. Behind the glove box is a 4 m-long vacuum path of polyvinyl chloride (PVC) tubing with an inner diameter of 83 mm. The second hutch is equipped with 1 and 2 m lengths of PVC tubing with 102 and 153 mm inner diameters, respectively. The wall separating the first and second hutches has a 102 mm inner diameter SUS nipple through the hutches. All components, including the glove box and the following PVC flight paths, are evacuated to reduce parasitic scattering, as shown schematically at the bottom of Fig. 1 ▸. The resulting AUSAXS system has a sample-to-detector distance of 9.1 m. The beam stopper is a tungsten rod with a width of 2 mm and an optical axis length of 4 mm. USAXS/AUSAXS profiles were obtained with a two-dimensional hybrid pixel array detector, PILATUS 300 K-W CdTe (DECTRIS Ltd), with 3 × 1 modules and 1475 × 195 pixels of 172 µm pixel size.

## 4. Performance

### 4.1. USAXS for silica particles

**Figure 2:**
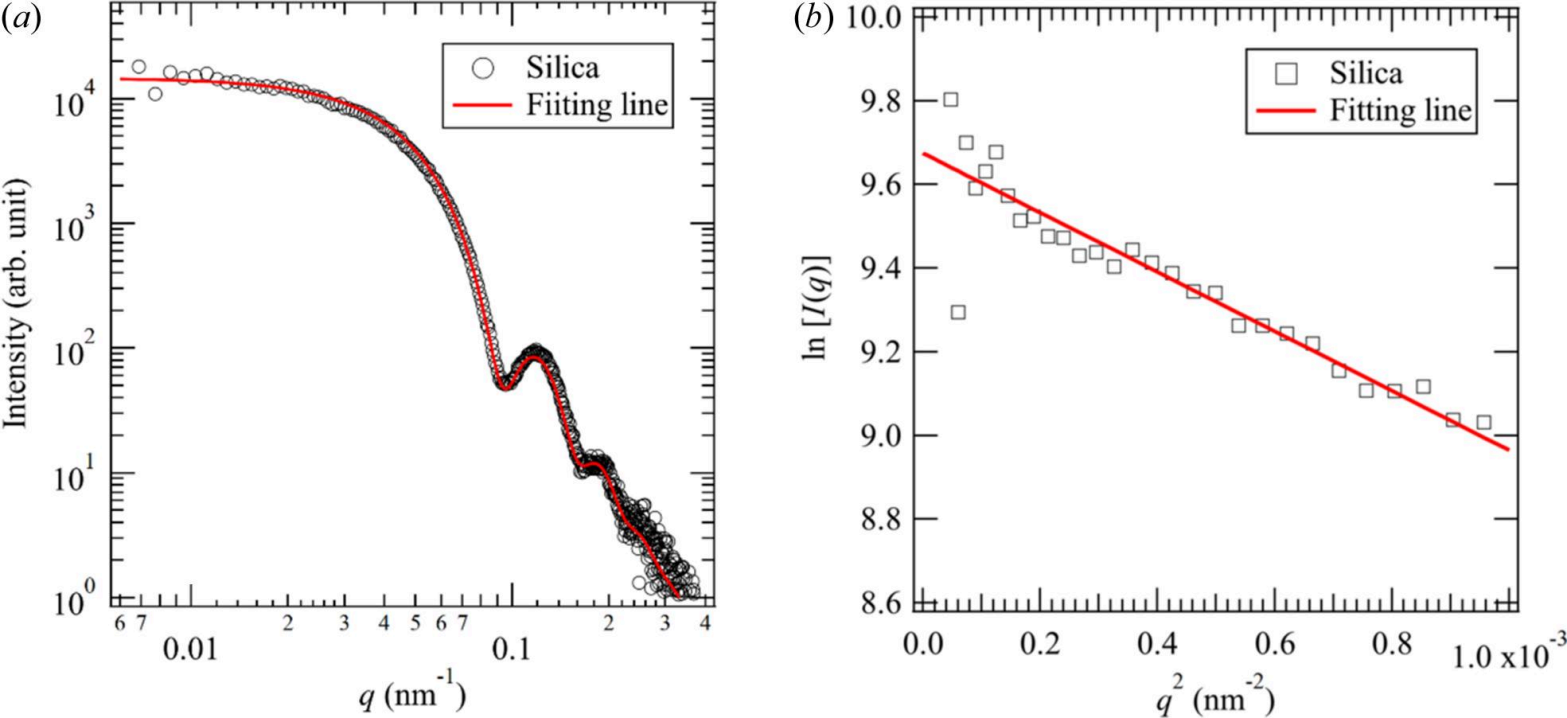
(*a*) USAXS profile of the SiP dispersion. (*b*) Guinier plot of the scattered intensity in the lower-*q* region.

To evaluate the minimum *q* obtained with the AUSAXS system, we measured a silica particle (SiP) dispersion. The SiP used in this measurement is monodispersed and its radius listed in the catalog is 50 nm. Fig. 2 ▸(*a*) shows a USAXS profile of the SiP dispersion with the energy of the incident beam being 9 keV. We observed the Guinier region in the lower-*q* region and several fringes representing the scattering of spheres. Fig. 2 ▸(*b*) shows a Guinier plot of the scattered intensity in the lower-*q* region. A linear relationship can be found up to *q* = 0.0069 nm^−1^, corresponding to the minimum *q* accessible with the AUSAXS system. The *z*-average radius *r*_0*z*_ estimated from the Guinier plot is 58.1 nm. We also evaluate the mean value of particle size *r*_0_ and distribution σ_0_ by fitting the scattered intensity at 0.0069 < *q* < 0.3 nm^−1^ with the scattering function for a sphere with a radius distribution (see details given in the supporting information). The fitting yielded the mean radius *r*_0_ = 46.5 nm and the standard deviation σ_0_ = 4.9 nm of the SiPs. The *z*-average radius *r*_0*z*, fitting_ estimated from the fitting is 47.5 nm, which is smaller than *r*_0*z*_ obtained from the Guinier plot, originating from the fact that the scattering function for fitting does not include the effects of the aggregation of the spheres. We list the characteristic parameters (*q* range and maximum flux value) at typical X-ray energies in Table 1 ▸. The minimum beam size is 0.1 × 0.1 mm, irrespective of the X-ray energy.

### 4.2. Time-resolved AUSAXS for vulcanized SBR

**Figure 3:**
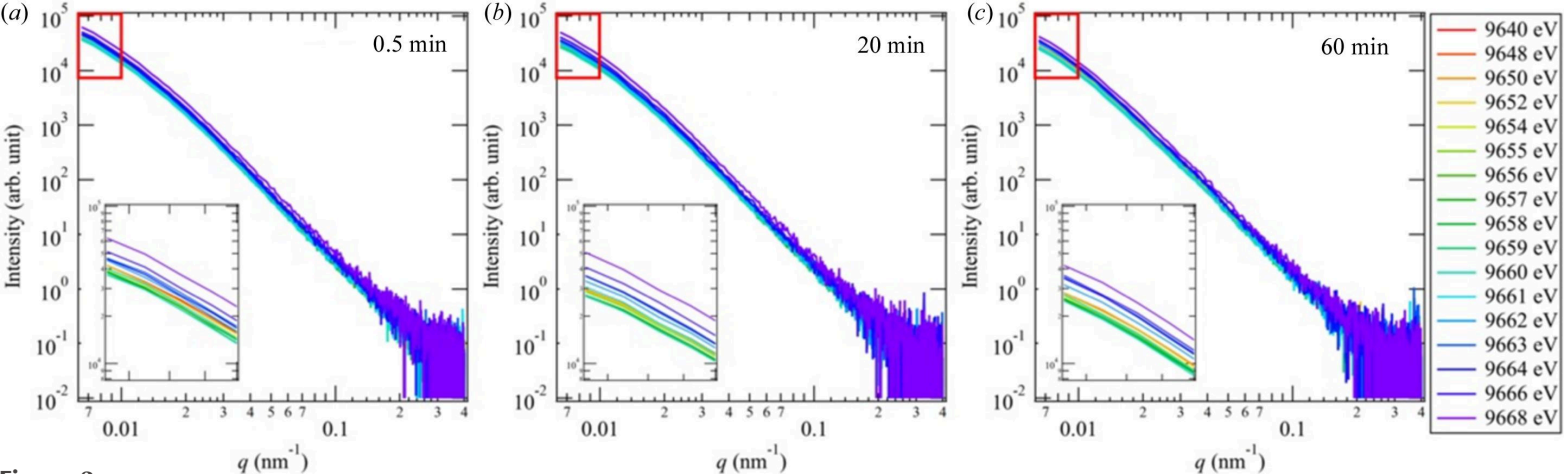
Time-resolved AUSAXS profiles during the vulcanization of SBR at 160°C at (*a*) 0.5 min, (*b*) 20 min and (*d*) 60 min. The insets are enlargements of the parts of the profiles in the smaller-*q* region indicated by red squares.

**Figure 4:**
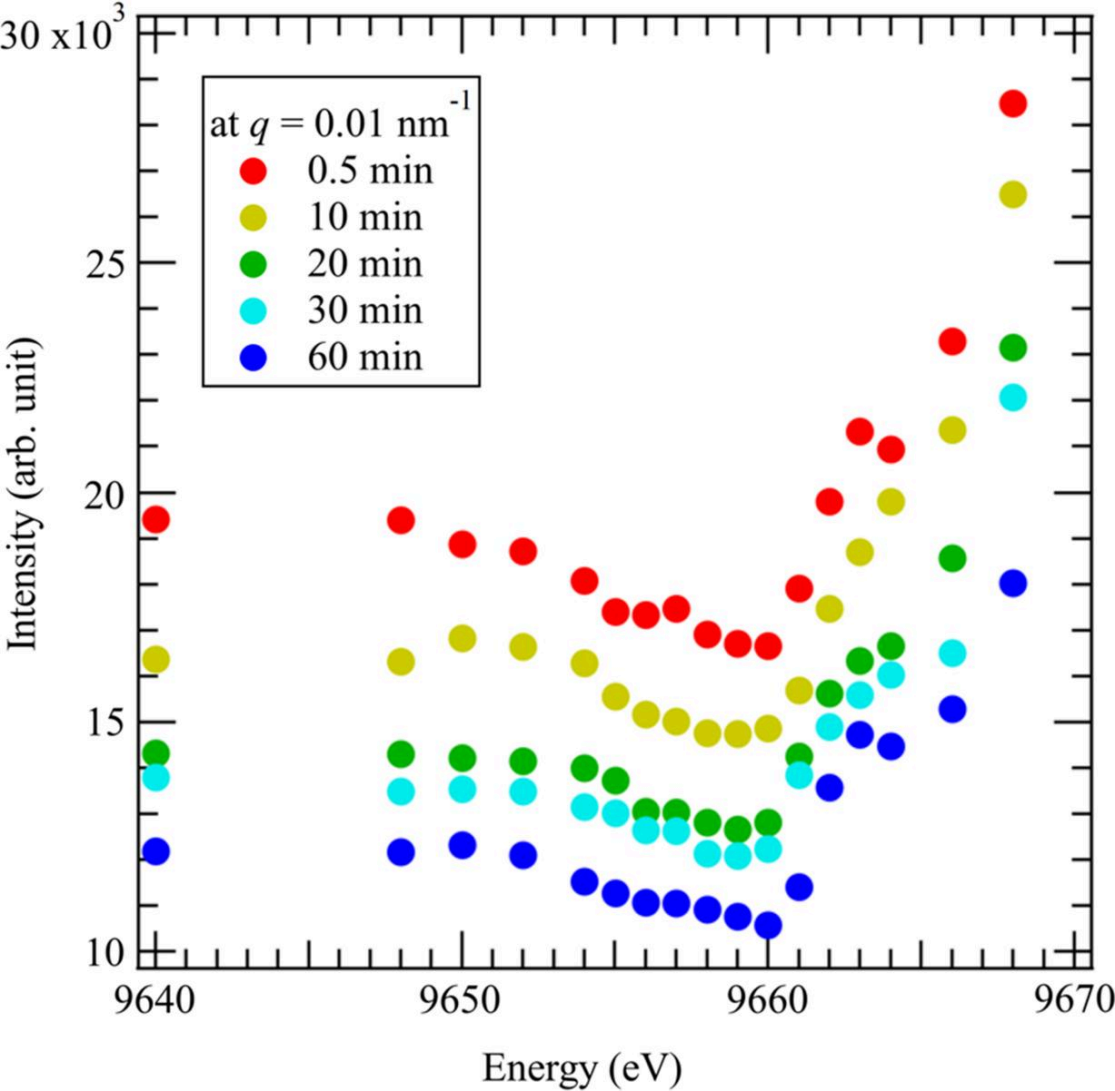
Change in the scattered intensity at 0.01 nm^−1^ with time as a function of X-ray energy.

As described in Section 3, we can quickly change the energy of the incident X-rays. To check the feasibility of time-resolved AUSAXS, we observed the change of ZnO structures with time during the vulcanization of rubber. It is well known that the addition of ZnO to rubber accelerates the vulcanization with sulfur and that the spatial distribution of ZnO affects the physical properties of vulcanized rubber. By applying time-resolved AUSAXS near the Zn *K* edge to the vulcanization of rubber, we can extract information on the change in the structures of ZnO during vulcanization. We used SBR as rubber. The details of the sample (SBR rubber) used for the measurement are given in the supporting information. Fig. 3 ▸ shows the time-resolved AUSAXS profiles during the vulcanization of the rubber. We measured the scattering profiles at the following 17 different energies in one cycle: 9640, 9648, 9650, 9652, 9654, 9655, 9656, 9657, 9658, 9659, 9660, 9661, 9662, 9663, 9664, 9666 and 9668 eV. Note that the energy of the Zn *K*-absorption edge of ZnO is 9660 eV. The scattering measurement for each energy is 1 s and each cycle takes 30 s. The scattered intensity varied with the energy of the X-rays in the lower-*q* region. In comparison, the scattered intensity in the higher-*q* region did not change much with the X-ray energy, indicating that the size of the ZnO structures is on the order of submicrometres. Fig. 4 ▸ shows the change in the energy dependence of the scattered intensity at *q* = 0.01 nm^−1^ and with time. Each plot shows the X-ray energy dependence, and a steep increase was found at 9660 eV, corresponding to the *K* edge of Zn. The change in the scattered intensity with X-ray energy near the *K* edge of Zn is caused by the change in the contrast factor of Zn. When the sample is treated as a Zn and SBR binary system, the scattered intensity *I*(*q*, *E*) at *q* and X-ray energy *E* can be described by

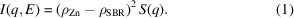

**Figure 5:**
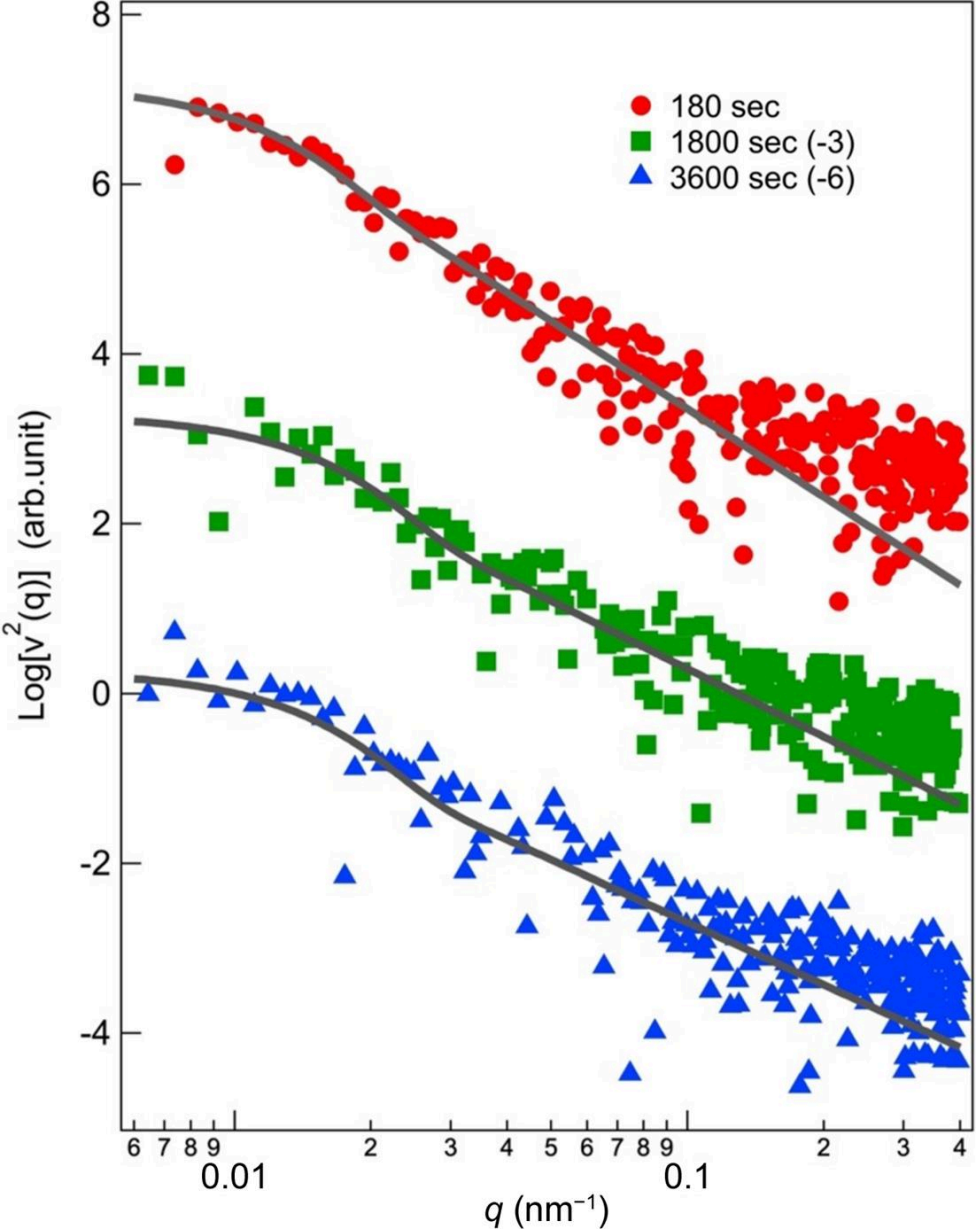
Change in ν^2^(*q*) with time plotted as a function of *q*. The shift of the plots is shown in the legend.

Here *S*(*q*) is the structure factor of the system and ρ_*i*_ is the scattering length density of the *i*th component. While ρ_SBR_ is almost independent of the X-ray energy in the experiment, ρ_Zn_ varies with the energy *E* of the incident X-rays since we conducted SAXS experiments near the Zn *K*-absorption edge. ρ_Zn_ is expressed by

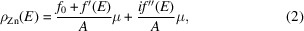

where *f*_0_, *A*, μ, *f*′(*E*) and *f*′′(*E*) are the atomic scattering factor, molar mass, specific gravity, and real and imaginary parts of the anomalous dispersion, respectively. *I*(*q*, *E*) is thus expressed by

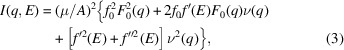

where *F*_0_(*q*) and ν(*q*) are the non-resonant and resonant amplitudes, respectively. We estimated ν^2^(*q*) from the scattered intensity below the *K* edge of Zn. We measured time-dependent data at a fixed energy for the entire reaction and checked whether the profiles change with time during the AUSAXS experiment. As shown in Fig. S1 of the supporting information, the profiles measured at a fixed energy did not change throughout the duration or 30 s at time *t* > 180 s, indicating that the system stayed in a steady state and hence we can analyze the data using equation (3). Fig. 5 ▸ shows the change in ν^2^(*q*) with *t*. We found a shoulder at *q* = 0.01 nm^−1^ in ν^2^(*q*), indicating that the clusters of ZnO are about 100 nm in the system. We fitted ν^2^(*q*) following the unified Guinier/power-law equation

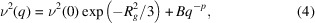

where ν^2^(0), *R*_g_, *B* and *p* are ν^2^(*q*) at *q* = 0, the radius of gyration of ZnO clusters, the prefactor of the power law and the exponent of the power law, respectively. The equation fits the experimental results well and the fitting results yielded the characteristic parameters listed in Table 2 ▸. The size of particles of ZnO and the exponent decrease with time, which is associated with the progress of the vulcanization. Further analyses will be reported in a forthcoming paper.

The X-ray energy dependencies of *f*′(*E*) and *f*′′(*E*) change with the chemical state of Zn and the energy dependence of the scattered intensity, thus reflecting the chemical state of Zn. We studied the change in the energy dependence of *I*(*q*, *E*) with time, which suggests that the transformation of ZnO into other compounds (*e.g.* zinc sulfide) by vulcanization (Ikeda *et al.*, 2019 ▸) takes place in the lower-*q* region.

## 5. Conclusions

We succeeded in constructing an AUSAXS measurement system by taking advantage of the BL28XU beamline at SPring-8. The *q* range for the AUSAXS measurement system was estimated to be 0.0069–0.3 nm^−1^ by measuring an SiP dispersion. We obtained the scattering profiles of an SBR sample at 17 different X-ray energies in 30 s and conducted time-resolved measurements to investigate the changes in the structure of zinc compounds in SBR rubber during vulcanization. A change in the energy dependence of *I*(*q*, *E*) with time was found during vulcanization, suggesting that the transformation of Zn can be attributed to the reaction. We plan to conduct AUSAXS measurements to clarify the changes in the structures and the transformation of the compounds during vulcanization.

Since we can conduct time-resolved AUSAXS measurements with the system, we can investigate the kinetics of the element-specific structure on the submicrometre scale. Thus we can apply this system to basic research on the association processes of metal ions in gelation and phase-separation dynamics of metal alloys, and on industrial materials including metals.

## 6. Related literature

The following reference is cited in the supporting information: Pedersen (1997 ▸).

## Supplementary Material

Supporting figure, table and equations. DOI: 10.1107/S1600576723011135/jl5077sup1.pdf

## Figures and Tables

**Table 1:** Characteristic parameters of the AUSAXS setup at BL28XU

X-ray energy (keV)	*q* range (nm^−1^)	Maximum flux [photons/(s mm^2^)]
6.000	4.6 × 10^−3^–2.0 × 10^−1^	8.174 × 10^10^
9.000	6.9 × 10^−3^–3.0 × 10^−1^	1.796 × 10^11^
30.000	2.3 × 10^−2^–1.0	2.465 × 10^10^

**Table 2:** Characteristic parameters obtained by fitting equation (4)

Time (s)	ν^2^(0)	*R*_g_ (nm)	*B*	*p*
180	1.50 × 10^7^	1.7 × 10^2^	8.1 × 10^−1^	3.4
1800	2.0 × 10^6^	1.3 × 10^2^	4.30	2.7
3600	1.9 × 10^6^	1.4 × 10^2^	7.1	2.4
