# Supplementary material for: Time-resolved AUSAXS at BL28XU at SPring-8
Source: J Appl Crystallogr. 2024 Feb 1;57(Pt 1):215–9. doi: 10.1107/S1600576723011135 (PMC10840312; doi:10.1107/S1600576723011135)
Supplement: Supplementary file 1 [file j-57-00215-sup1.pdf]

JOURNAL OF APPLIED CRYSTALLOGRAPHY

Volume 57 (2024)

Supporting information for article:

Time-resolved AUSAXS at BL28XU at SPring-8

Yohei Nakanishi, So Fujinami, Motoki Shibata, Tsukasa Miyazaki, Katsuhiro Yamamoto and Mikihito Takenaka

S1. Materials

Silica particles (SiPs, sicastar 43-00-102; micromod Partikeltechnologie GmbH, Rostock, Germany) were dispersed in distilled water, and the obtained 0.5 wt% of SiP dispersion was placed in a solution cell with a 20 μm thick of quartz glass windows. The particle size and polydispersity index of SiPs in the catalog were 100 nm and <0.2, respectively. Poly (styrene-*ran*-butadiene) (SBR) (JSR1502; JSR Corporation, Japan) were used as the rubber materials. Zinc oxide (ZnO; Seido Chemical Industry Co., Ltd, Japan), stearic acid (FUJIFILM Wako Pure Chemical Corporation, Japan, 95.0%), sulfur (325 mesh, Hosoi Chemical Industry Co., Ltd, Japan, 99.9%), and *N*-*t*-butyl-2-benzothiazole sulfonamide (TBBS, Sanceler NS; Sanshin Chemical Industry Co., Ltd., Japan) were used as received. Rubber sample sheets with a thickness of 1.5 mm were prepared according to Table 3. The SBR samples were inserted into the heater at 160°C for vulcanization.

Table 3. Composition of the SBR rubber for AUSAXS.

Component	SBR	ZnO	Stearic acid	Sulfur	TBBS
Weight ratio	100	3	3	2	1.5

S2. Scattering model for the SiP dispersion

The X-ray scattering intensity $I(q)$ of the dilute solution of particles can be described by the following equation:

$$I(q) = n(\Delta\rho_e)^2 \langle F(q)^2 \rangle \tag{5}$$

where n is the number density of SiP particles, $\Delta\rho_e$ is the difference in the scattering length density between a particle and solvent, and $F(q)$ is the form factor of particles. Considering the polydispersity of the SiP particles, $\langle F(q)^2 \rangle$ are given by (Pedersen, 1997)

$$F(q) = \frac{4\pi r^3[\sin(qr) - qr\cos(qr)]}{(qr)^3} \tag{6}$$

$$\langle F(q)^2 \rangle = \int_0^\infty F(q)^2 D(r)\, dr \tag{7}$$

where $D(r)$ is the Gaussian distribution function with the average radius r_0 and its standard deviation σ_0 of the particle. $D(r)$ is given by

$$D(r) = \frac{1}{\sqrt{2\pi}\sigma_0} \exp\left[-\frac{(r - r_0)^2}{2{\sigma_0}^2}\right] \tag{8}$$

S3. Change in the scattered intensity with time at a given energy

Since we need the collection of data at multiple energies the system should be either in a steady state in terms of changes in structures. Thus, we have checked whether the scattered intensity at a given energy did not change during duration time 30 sec of AUSAXS measurements. Figure 6 shows the time change of the scattered intensity at 9640 eV during duration time for 180 sec and 1800 sec. The scattered intensity did not change during duration time. Thus, we can analyze the data with Eq.(3).

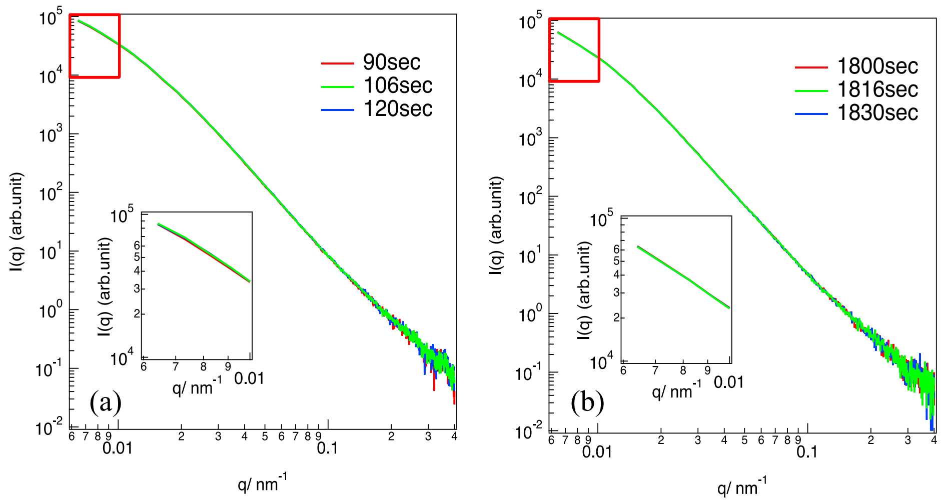

Figure S1 Time changes of the scattered intensity at 9640 eV during duration time for (a)180 sec and (b) 1800 sec. Insets are enlarged parts of the profiles at smaller q-region indicated by red squares.
